# Lipocalin 2 (LCN2) Expression in Hepatic Malfunction and Therapy

**DOI:** 10.3389/fphys.2016.00430

**Published:** 2016-09-27

**Authors:** Anastasia Asimakopoulou, Sabine Weiskirchen, Ralf Weiskirchen

**Affiliations:** Institute of Molecular Pathobiochemistry, Experimental Gene Therapy and Clinical Chemistry, RWTH University Hospital AachenAachen, Germany

**Keywords:** inflammation, liver, biomarker, alcoholic fatty liver disease, non-alcoholic steatohepatitis, matrix metalloproteinases, liver failure, hepatic disease

## Abstract

Lipocalin 2 (LCN2) is a secreted protein that belongs to the Lipocalins, a group of transporters of small lipophilic molecules such as steroids, lipopolysaccharides, iron, and fatty acids in circulation. Two decades after its discovery and after a high variety of published findings, LCN2's altered expression has been assigned to critical roles in several pathological organ conditions, including liver injury and steatosis, renal damage, brain injury, cardiomyopathies, muscle-skeletal disorders, lung infection, and cancer in several organs. The significance of this 25-kDa lipocalin molecule has been impressively increased during the last years. Data from several studies indicate the role of LCN2 in physiological conditions as well as in response to cellular stress and injury. LCN2 in the liver shows a protective role in acute and chronic injury models where its expression is highly elevated. Moreover, LCN2 expression is being considered as a potential strong biomarker for pathological conditions, including rheumatic diseases, cancer in human organs, hepatic steatosis, hepatic damage, and inflammation. In this review, we summarize experimental and clinical findings linking LCN2 to the pathogenesis of liver disease.

## Introduction

LCN2 has many synonyms including neutrophil gelatinase associated lipocalin (NGAL), 24p3, oncogenic lipocalin, siderocalin, 25 kDa α_2_-microglobulin-related protein, and uterocalin. The different names are justified by predicting functions of LCN2 or tissue in which the expression was first detected. LCN2 belongs to the broad family of lipocalins that were first proposed to have unifying functions in the transport of hydrophobic substances. This ever expanding family of proteins share limited regions of sequence homology and a common tertiary structure architecture. Details on the structure, characteristics, and most known functions of members of the lipocalin family were highlighted in a previous report (Asimakopoulou and Weiskirchen, 2015).

Human LCN2 was initially isolated and purified by Kjeldsen and coworkers as a 25-kDa neutrophil protein (Table 1), that is in part associated with gelatinase from human neutrophils (Kjeldsen et al., 1993). One year later, the same group cloned the full length human LCN2 cDNA and showed that this lipocalin is mainly expressed in myeloid cells (Bundgaard et al., 1994) and later assigned to a cluster of at least three lipocalins on the long arm of human chromosome 9 by *in situ* hybridization that is syntenic to a region of mouse chromosome 4 (Chan et al., 1994).

**Table 1:** **Compilation of data on structure and function of LCN2**.

Discovery	LCN2 was first purified and identified from human phorbol myristate acetate-stimulated neutrophils as a gelatinase-associated protein.
Terminology	Acronyms for human LCN2 are: Neutrophil gelatinase-associated Lipocalin (NGAL), human neutrophil lipocalin (HNL), 25 kDa α2-microglobulin-related protein, and Siderocalin. Further acronyms are oncogene 24p3 protein (24p3), Uterocalin, and 24 kDa superinducible protein (Sip24) in mouse and Neu-related lipocalin (NRL) and α2u-globulin in rat.
Gene structure/chromosomal localization	LCN2 is localized on the long arm of chromosome 9, (9q34.11) in a cluster of at least three lipocalins; the primary transcript is 3696-bp encompassing 7 exons and 6 introns.
Protein structure	LCN2 is a secreted ~25 kDa protein composed of 178 amino acids and belonging to the lipocalin family which transport small hydrophobic molecules. These proteins share a similar three-dimensional fold composed of an eight-stranded, antiparallel symmetrical β-barrel. One end of the barrel is open providing access to the binding site within the barrel cavity. LCN2 occurs in monomeric and dimeric forms.
Biosynthesis/expression	The steady state mRNA levels of LCN2 are high in bone marrow but not in peripheral leukocytes. High levels of LCN2 are found in tissue (e.g., uterus, prostate, salivary gland, stomach, appendix, colon, trachea, and lung) that are often exposed to microorganisms or glands that secrete to such tissues.
Functions	Secreted transport protein, siderophore, modulator of innate immune response, binding partner of human gelatinase (MMP-9) and bacterial catecholate-type ferric siderophores, delivers iron to the cytoplasma and acts as an activator/repressor of iron-responsive genes.
Regulation	LCN2 is an acute phase protein that is substantially activated by lipopolysaccharides and activated by the inducible transcription factor NF-κB. Moreover, dexamethasone and retionoic acid increase expression of LCN2. Selected cytokines reported to induce LCN2 expression are IL-6, IL-1β, IL-10, IL-17, TNF-α, and TGF-α.
Receptors	Actually two LCN2 receptors are known, namely the low density lipoprotein-related protein 2 (LRP2, Megalin) and NGALR2 (solute carrier family 22 member 17, SLC22A17, or 24p3R).
Target genes	Transient overexpression experiments showed that LCN2 modifies expression of mesenchymal markers (e.g., vimentin, fibronectin), E-cadherin, and the lipid droplet-associated protein PLIN5 (OXPAT).
Clearance/half life	~10 min (monomeric form) and ~20 min (dimeric form).
Presence in body fluids	LCN2 is detectable in serum, plasma, urine, and tissue extracts.
Pathobiochemistry	Increased expression during inflammatory activity in many organs (e.g., kidney, heart, brain, pancreas, lungs, and liver).
Animal models	*Lcn2*-deficient mice in which exons 1–5 or exons 2–5 were replaced are viable but exhibit increased sensitivity to *E. coli* infections and lipopolysaccharides showing that LCN2 is an important component of the innate immune systems and the acute phase response. It limits bacterial growth by sequestering iron-laden siderophores. Respective mice show alterations in intracellular lipid droplet formation.

*The depicted information was taken from Triebel et al. (1992), Kjeldsen et al. (1993), Bundgaard et al. (1994), Axelsson et al. (1995), Garay-Rojas et al. (1996), Cowland and Borregaard (1997), Goetz et al. (2002), Yang et al. (2002), Flo et al. (2004), Cai et al. (2010), Yang et al. (2009), and Asimakopoulou and Weiskirchen (2015)*.

**Figure 1:**
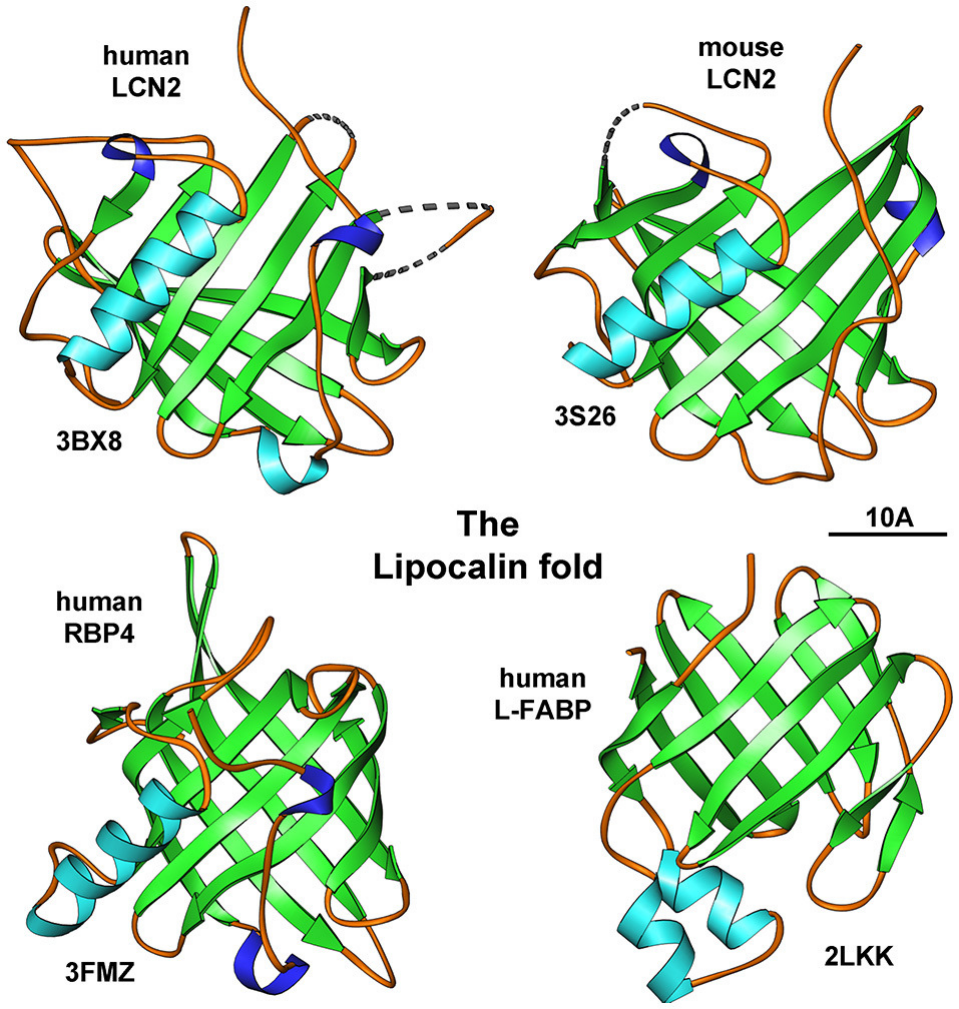
**The lipocalin fold**. Members of the lipocalin family have a typical eight-stranded, anti-parallel, symmetrical β-barrel fold structure. Depicted are human and mouse LCN2, human retniol-binding protein 4 (RBP4), and human liver fatty acid-binding protein (L-FABP). The depicted structures were generated using the Ribbons XP software (version 3.0) and coordinates 3BX8, 3S26, 3FMZ, and 2LKK deposited in the RCSB Protein Data Bank (http://www.rcsb.org). A size marker (10 Å) is given.

The first critical roles given to LCN2 were proposed from its “lipocalin structure” that is most closely related to the structures of the epididymal retinoic acid-binding proteins and the major urinary protein (Goetz et al., 2000). The typical unifying three dimensional fold of lipocalins encompasses an eight-stranded, anti-parallel, symmetrical β-barrel fold with a cylindrical shape (Figure 1).

Later it was shown that murine and human LCN2 are both iron-trafficking proteins that interfere with the activity of iron-responsive genes (Yang et al., 2002). The initial finding that LCN2 is involved in iron delivery pointed to a potential key role of LCN2 in immunity. This assumption was further confirmed in experiments showing that LCN2 has the capacity to limit bacterial growth by sequestering the iron-laden siderophore after infection in mice (Flo et al., 2004; Holmes et al., 2005; Berger et al., 2006). After this very first allocation of LCN2 as a significant factor in guaranteeing immune balance, the repertoire of LCN2 functions was further expanded to many biological processes, including inflammation, intoxication, immune defense, organogenesis, and cancer.

In this review, we will summarize some of the major findings of LCN2 in the pathogenesis of organ disease with a special emphasis on inflammatory liver diseases.

## Regulation of LCN2

LCN2 starts being expressed in the embryonic stage (Mallbris et al., 2002) and it has been shown to be activated strongly in inflamed organs such as liver, heart, lungs, bone marrow, kidney, and spleen (Aigner et al., 2007; Borkham-Kamphorst et al., 2011). LCN2 expression is also induced during neutrophil maturation (Kjeldsen et al., 1993). It has been demonstrated to decrease at an older age, especially in the kidney and liver. Further, LCN2 is dramatically increased in cells by addition of dexamethasone and retinoic acid (Garay-Rojas et al., 1996). During bacterial infection, the expression of LCN2 has been proved to be upregulated in several tissues such as liver (Xu et al., 2015), diverse mucosal tissues (Kjeldsen et al., 2000), lung (Chan et al., 2009), and epithelial tissue (Friedl et al., 1999). In all these tissues LCN2 behaves as an acute phase protein. At sites of infection LCN2 is secreted by neutrophils, macrophages, activated leukocytes, and adipocytes (Kjeldsen et al., 1993; Meheus et al., 1993; Flo et al., 2004). The different reports unanimously show that LCN2 is either increased in the tissue by several resident cell types or alternatively by circulating immune cells that are recruited into the inflamed tissue.

In *in vitro* and *in vivo* experimentation, LCN2 was found to be induced by several factors such as lipopolysaccharide (LPS), cytokines, retinoic acid, growth factors, and insulin (Meheus et al., 1993; Sorensen et al., 2003; Bu et al., 2006; Sunil et al., 2007; Tan et al., 2009; Hamzic et al., 2013). LPS has been shown to be a strong inducer of LCN2 in liver and lungs (Sunil et al., 2007; Borkham-Kamphorst et al., 2013). The stimulatory capacity of LPS in mice is extremely high when compared to other hepatotoxins such as carbon tetrachloride and Concanavalin A (Borkham-Kamphorst et al., 2013). There are several other biomolecules and pathways known to contribute to the expression of LCN2 in inflammatory cells, macrophage cell lines, epithelial cells, breast cancer cell lines, and hepatocytes (Meheus et al., 1993; Seth et al., 2002; Bu et al., 2006; Borkham-Kamphorst et al., 2011; Kienzl-Wagner et al., 2015; Xu et al., 2015). Cytokines that induce the expression of LCN2 include the interleukins IL-6, IL-1β, IL-10, IL-17, tumor necrosis factor (TNF-α), and tumor growth factor (TGF-α) (Sorensen et al., 2003; Bu et al., 2006; Borkham-Kamphorst et al., 2011; Hamzic et al., 2013; Vazquez et al., 2015; Xu et al., 2015). The nuclear factor-κB (NF-κB) transcription activity seems to be the main pathway involved in LCN2 stimulation (Bu et al., 2006). The close linkage to the NF-κB pathway and diverse pro-inflammatory cytokines that act through the activation of NF-κB was also found in different models of hepatic injury (Borkham-Kamphorst et al., 2011).

Up to date, LCN2 has been found to bind only on two receptors. Hvidberg and colleagues first described that LCN2 binds to Megalin, representing a member of the low-density lipoprotein receptor family (Hvidberg et al., 2005) while another study described a 24p3R receptor of LCN2 that confers the ability to undergo either iron uptake or apoptosis (Devireddy et al., 2005). Although the concept that LCN2 does induce iron efflux or stimulate apoptosis was somewhat challenged (Correnti et al., 2012), a more recent fundamental study performed with human homolog LCN2R (also known as SLC22A17) demonstrated that the N-terminal part of this receptor has a soluble extracellular domain that is intrinsically disordered with the capacity to preferentially interact with LCN2 in its non-iron bound form suggesting that this cellular receptor might discriminate between the iron-free (apo-) and iron-bound (holo-) form of LCN2 (Cabedo Martinez et al., 2016).

## LCN2 pathophysiological expression in tissue

**Figure 2:**
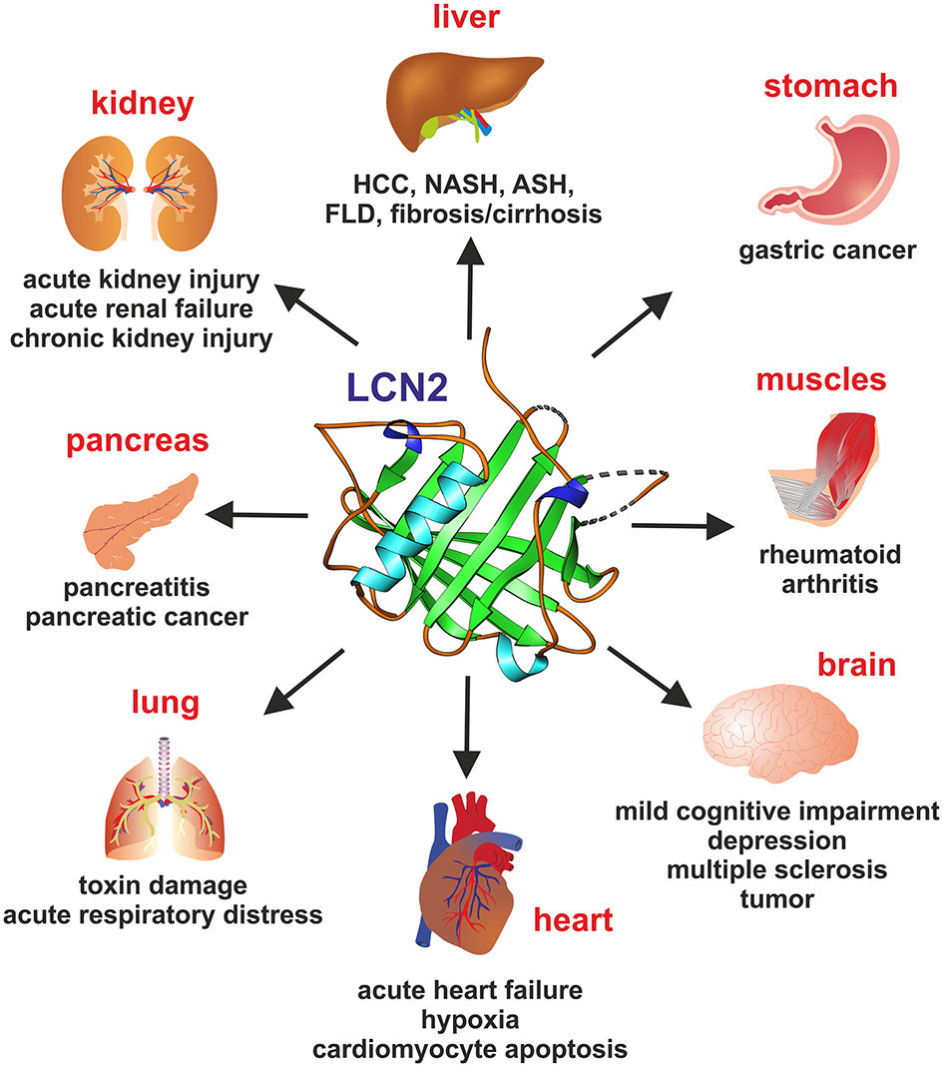
**Lipocalin 2 (LCN2) in organ damage**. Representative pathophysiological or malignancies conditions for which elevated LCN2 concentrations in representative organs are indicative are depicted.

LCN2 is reported to be expressed in numerous pathological and experimental-induced conditions in many organs and tissues such as liver, kidney, lungs, bone, brain, and heart (Figure 2). Several of the published findings on main organs are listed in Table 2.

**Table 2:** **Selected experimental and clinical findings associated with altered LCN2 expression in organ disorders**.

**Organ**	**Species**	**Model/disease**	**Method**	**LCN2 expression**	**Finding**	**References**
Kidney	Mouse/human	Chronic kidney disease/proteinuria	qRT-PCR, WB, IHC	Increased LCN2 expression	Induction of Ca^2+^-dependent ER stress causes albumin to activate ATF4 expression, which in turn induces LCN2 production leading to increased apoptosis and tubulointerstitial damage	El Karoui et al., 2016
	Mouse	Renal allograft damage following transplantation	ELISA, qRT-PCR	Strong upregulation of LCN2	LCN2 improved morphological and functional damage by reducing apoptosis of tubular epithelial cells and stimulating their proliferation	Ashraf et al., 2016
	Mouse	Antibody-induced glomerulonephritis	IHC, ELISA	LCN2 expression is increased in mice with nephrotoxic nephritis	Renal binding of pathogenic antibodies stimulates LCN2 expression	Pawar et al., 2012
	Human	Acute kidney injury (AKI)	ELISA	Strong upregulation of LCN2 in progressive AKI	Urinary high LCN2 expression indicates risk for progression and death of AKI patients	Belcher et al., 2014
	Human newborns	Acute kidney injury/failure	ELISA, WB	Upregulation of LCN2 in serum and blood	LCN2 is a diagnostic marker for AKI in neonates	Jin et al., 2011
Heart	Human/children	Acute heart failure/worsening renal function	ELISA	High plasma LCN2 concentration	Admission plasma LCN2 level can predict worsening renal function in children hospitalized for acute heart failure	Elsharawy et al., 2016
	Mouse, rat	Heart failure/cardiomyocyte apoptosis	NA	NA	Recombinant LCN2 directly induces cardiomyocyte apoptosis which occurs via a mechanism involving elevated intracellular iron levels	Xu et al., 2012
	Mouse	Hypoxia-induced cardiomyocyte apoptosis	qRT-PCR, WB	Transient overexpression by transfection	Induced up-regulation of miR-138 inhibits the hypoxia-induced cardiomyocyte apoptosis via down-regulating expression of pro-apoptotic LCN2; LCN2 is a direct target of miR-138	Xiong et al., 2016
Brain	Human	Mild cognitive impairment (MCI) and Alzheimer's disease (AD)	ELISA	LCN2 levels significantly higher in MCI patients compared to the healthy control	Increased plasma LCN2 levels during MCI could be helpful in predicting the progression from MCI to AD	Choi et al., 2011
	Human	Depression	ELISA	Elevated plasma LCN2 levels in depressed subjects	LCN2 plasma levels are increased in depressed older persons, resistant to antidepressant medication and age; plasma LCN2 is a possible biomarker for late-life depression	Naudé et al., 2013
	Mouse/human	Multiple sclerosis (MS)	IF, IHC, ELISA, qRT-PCR	LCN2 expression strongly induced after murine MS model and in human blood-cerebrospinal fluid samples of MS patients	LCN2 a valuable molecule for the diagnostics and monitoring of MS	Marques et al., 2012
Pancreas	Human	Pancreatitis	ELISA	Urinary LCN2 levels associated with increased severity and mortality	LCN2 a promising diagnostic and prognostic factor for severe acute pancreatitis in an early stage of the disease	Lipinski et al., 2015
	Human	Chronic pancreatitis, pancreatic cancer	ELISA	LCN2 significantly elevated in the pancreatic juice of patients	LCN2 could help establish etiology for pancreatitis	Kaur et al., 2013
Lung	Mouse	LPS-induced acute lung injury	ELISA, WB, qRT-PCR	LCN2 levels upregulated in diseased mice	LCN2 is a promising in detection of experiment acute lung injury	Zeng et al., 2014
	Human	Sepsis-induced acute respiratory distress syndrome	qRT-PCR	Significantly elevated LCN2 mRNA expression	LCN2 with other neutrophil related genes participates in neutrophil-related mechanisms in progression to acute respiratory distress syndrome	Kangelaris et al., 2015
Musculo-skeletal tissues	Human	Rheumatoid arthritis	ELISA	LCN2 was highly expressed in the synovial fluids of patients compared to healthy controls	LCN2 is a possible diagnostic marker for rheumatoid arthritis	Bläser et al., 1995
Skin	Mouse	Topical psoriasiform skin	NA	LCN2 enhances expression of Th17 cytokines/chemokines and antimicrobial peptides	LCN2 potentiates the development of psoriasis	Hau et al., 2016
Cancer	Human	Brain tumor	Zymography, ELISA, IHC	Great increase of LCN2 in tumor tissue and clearing after tumor resection	Useful predictor of the presence of brain tumors	Smith et al., 2008
	Human	Gastric cancer	MMP-9/LCN2 complex zymography, WB, IHC, IF	Enhance expression of MMP-9/LCN2 in cancer tissue	Enhanced levels of MMP-9/LCN2 complex is highly prognostic for worse survival in gastric cancer patients	Kubben et al., 2007
	Human	Pancreatic adenocarcinoma	IHC, ELISA, qRT-PCR, WB	LCN2 overexpression in pancreatic cancer lesions	LCN2 might participate in pancreatic cell transdifferentiation and progress of cancer	Moniaux et al., 2008
	Human	HCC	IHC	Upregulated LCN2 expression in HCC significantly correlated with unfavorable clinicopathologic features	LCN2 could serve as prognostic factor and potential therapeutic target in HCC	Zhang et al., 2012
	Human	Endometrial cancer	IHC	LCN2 expression was associated with VEGF-A expression and distant tumor recurrences	LCN2 expression is associated with aggressive features and poor prognosis of endometrial cancer	Mannelqvist et al., 2012
	Human	Breast cancer	IHC, ELISA	Elevated LCN2 levels were found at advanced breast cancer stages in breast tissue and urine	LCN2 promotes breast cancer progression by inducing EMT	Yang et al., 2009
	Mouse	Breast cancer model and cell culture models	ELISA	LCN2 contributes to early events in metastasis, induces EMT, enhances migration and invasion	Tumor stroma-derived LCN2 promotes breast cancer metastasis	Ören et al., 2016

*AD, Alzheimer's disease; AKI, acute kidney injury; ELISA, Enzyme-linked immunosorbent assay; EMT, epithelial to mesenchymal transition; IF, immunofluoresence; IHC, immuohistochemistry; MCI, mild cognitive impairment; MS, Multiple sclerosis; qRT-PCR, quantitative real time polymerase chain reaction; WB, Western blot*.

Here we will first focus on some of the most recent reports on LCN2 expression and organ disorders to understand the possible major functions of LCN2 and subsequently highlight the importance of LCN2 in the pathogenesis of hepatic disease.

During the last years, LCN2 has been well-studied as a potential biomarker for kidney injury or to estimate the outcome of respective diseases (Coca et al., 2008; Heyne et al., 2012; Paragas et al., 2012; Barreto et al., 2014; Matsa et al., 2014). In one multicenter, prospective cohort study, a significant correlation between urinary injury biomarkers (LCN2, IL-18, KIM-1, L-FABP, and albuminuria) and disease outcome was found in which LCN2 only distinguished progression alone from no progression, suggesting that LCN2 as other proteins with similar behavior could potentially serve to identify patients at highest risk of progression and death (Belcher et al., 2014). More recently a systematic review and meta-analysis on LCN2 in acute kidney injury (AKI) or failure investigating more than 50 studies concluded that blood LCN2 could be used as a diagnostic marker for AKI in newborns (Jiang and Cui, 2015). Moreover, several investigators have suggested urinary LCN2 as an early diagnostic marker in renal impairment and AKI in chronic cirrhosis (Gerbes et al., 2011; Fagundes et al., 2012; Verna et al., 2012; Wong and Murray, 2014).

In addition to acute injury in the kidney, another study reported an active critical role of LCN2 activity in the formation of chronic kidney disease (Viau et al., 2010). In this report, it was found in experimental models of chronic kidney disease in mice from different genetic backgrounds, that the severity of renal lesions was strongly dependent on LCN2 that significantly correlated with hyperproliferation and disease progression in both mice and humans. Moreover, the authors found that LCN2 mediates the mitogenic effects of EGFR that critically predicts the severity of renal lesions after nephron reduction. These findings gave LCN2 a critical role in the pathogenetic pathways that lead to progressive renal failure and cystogenesis (Viau et al., 2010). In extension to their observation, the same group recently showed that ATF4 is mandatory for the efficient induction of LCN2 in the course of albumin-induced tubular damage (El Karoui et al., 2016). In this condition, the high concentration of LCN2 induces endoplasmic reticulum stress-induced apoptosis leading to chronic kidney disease, proteinuria and severe tubulointerstitial damage, which ultimately leads to end-stage renal disease (El Karoui et al., 2016). The authors proposed a model of tubulointerstitial injury caused by proteinuria where during endoplasmic reticulum (ER) stress, albumin activates the activating transcription factor 4 (ATF4) which in turn induces LCN2 production that leads further to apoptosis and tubulointerstitial damage. Most interestingly, providing mouse models and human clinical data, it was conclusively proved that 4-phenylbutyric acid that is used as a drug in humans to allow the kidneys to excrete excess nitrogen, has the ability to reduce ER stress thereby preventing proteinuria-induced renal lesions and LCN2 overexpression. The inhibition of LCN2 in this pathway reduced mortality and protected kidneys from degradation, morphological and functional (El Karoui et al., 2016). Similarly, a crucial role of LCN2 was proven in a mouse model of nephrotoxic nephritis in which the injection of LCN2 promoted inflammation, apoptosis, and exacerbated nephritis (Pawar et al., 2012).

In contrast to these findings, another study investigating acute allograft rejection in mice, showed that the treatment with recombinant LCN2 after transplantation reduced the extent of kidney allograft rejection, renal allograft damage, apoptosis of tubular epithelial cells, and induced their proliferation. The proliferation led to an amelioration of damage morphology and improved allograft function (Ashraf et al., 2016). These two studies paradigmatically demonstrate that LCN2 is a versatile biomolecule with outstanding pathophysiological, diagnostically, and potential therapeutic relevance.

The heart and kidney have numerous similarities and their relationship makes it understandable that renal dysfunction/failure often accompanies cardiac dysfunction/failure and the opposite (Shah and Greaves, 2010). Except the significant role that LCN2 seems to play in renal disorders, studies on cardiomyopathies indicate a wider role of LCN2. So far it was known that acute heart failure is frequently associated with worsening renal function in adult patients. Although investigations on the role of LCN2 in the heart have only recently begun, a very recent report showed that plasma LCN2 levels can also predict worsening renal function in hospitalized children with acute heart failure (Elsharawy et al., 2016). Mechanistically, a recent review on cardiomyopathies discusses how LCN2 can regulate heart failure based on iron overload and iron deficiency (Chan et al., 2015). The authors highlight that LCN2 is a very critical factor involved in regulating various cellular mechanisms including oxidative stress, mitochondrial dysfunction, ER stress, and autophagy that all can contribute to cardiac dysfunction when perturbed and optimal iron levels are absent (Chan et al., 2015).

Based on the role of LCN2 in iron transport, it was also speculated that circulating LCN2 levels may reflect the body's iron status in haemodialysis patients (Bolignano et al., 2009). LCN2 leads to cardiomyocyte apoptosis by causing intracellular iron accumulation (Xu et al., 2012). On the other side, the up-regulation of the miR-138 gene inhibits the hypoxia-induced cardiomyocyte apoptosis via down-regulating of LCN2 acting pro-apoptotic (Xiong et al., 2016). All these data indicate that LCN2 is a very sensitive regulator of cell survival and apoptosis in cardiomyocytes. In addition, these strong indications of LCN2's correlation to heart disorders makes further elucidation of LCN2 role in heart failure a very interesting and crucial topic to study.

Several conflicting results on the role of LCN2 in neurological processes are published. Some studies point to its neurodeleterious effects, while others indicate neuroprotection. In a clinical study that enrolled 41 patients with mild cognitive impairment (MCI), 62 patients with Alzheimer's disease and 38 healthy elderly control subjects, it was found that MCI, which is a prodromal-inflammation stage of Alzheimer's disease, is correlated with elevated plasma LCN2 levels (Choi et al., 2011). Depression was also associated with LCN2 plasma levels in a cohort of 350 depressed persons that were compared to 129 non-depressed older subjects (Naudé et al., 2013). The study further revealed that the concentration of LCN2 was higher in patients with a recurrent depression compared to those with a first episode and independent of antidepressant treatment. A study performed in an experimental model of multiple sclerosis (MS) in mice and also analyzing cerebrospinal fluid samples taken from human patients suffering from MS showed increased LCN2 levels in the active phases of experimental autoimmune encephalomyelitis and in MS patients when compared to healthy individuals. Remarkably, the increase of LCN2 levels in the experimental animal model was reverted by the humanized monoclonal antibody Natalizumab that in humans is used for MS treatment supporting the notion that LCN2 measurement should be included in the diagnostic panel of markers for MS (Marques et al., 2012). However, the precise impact of LCN2 in the brain is still in the very beginning and further studies are required to unravel its roles in the nervous system.

Additionally, LCN2 has been studied in the endocrine gland disorders. In 2013 a study had strongly suggested LCN2 in the alkaline pancreatic juice as a marker for differentiating diseased from non-diseased pancreas and that LCN2 could contribute to understanding the etiology of pancreatitis (Kaur et al., 2013). Similarly, a study investigating the urinary levels of LCN2 in a cohort of more than 100 patients with inflammation of the pancreas found that the levels of LCN2 correlated with the severity of acute pancreatitis and mortality rate indicating that LCN2 could also be useful as biomarker for acute pancreatitis (Lipinski et al., 2015).

Preliminary studies performed in mice also point to a diagnostic role of LCN2 in LPS-induced septic acute lung injury (Zeng et al., 2014). In the same line, an investigation analyzing early sequence of events leading to the development of the acute respiratory distress syndrome, representing a common complication of sepsis found that the mRNA levels of LCN2 and other genes involved in the initial neutrophil response are upregulated in acute respiratory distress syndrome in respective patients (Kangelaris et al., 2015). These studies on LCN2 show that analyzing functional aspects of this lipocalin could also help to understand the pathogenesis or progression of lung diseases.

Moreover, strong indications of functions of LCN2 have been identified in musculoskeletal diseases. Bläser and colleagues reported in 1995 that very high concentrations of LCN2 were found in the synovial fluids (viscous fluid present in the cavities of movable joints) of patients suffering from inflammatory rheumatoid arthritis (Bläser et al., 1995). LCN2 was also proposed in a study analyzing a murine model of cartilage degradation a biomarker to estimate the severity of degeneration and inflammation (Wilson et al., 2008). In addition, LCN2 transgenic mice were marked by a smaller phenotype, presented bones micro-architectural changes in both endochondrial and intramembranous bones, and further showed a reduced deposition in the osteoblast bone matrix and impairment in expansion of bone marrow cavity (Costa et al., 2013).

Dysbalanced LCN2 expression has also been associated with cancer formation and progression in several organs. According to numerous studies in which LCN2 was quantitated by immunological methods such as immunohistochemistry, ELISA, gel zymography, and Western blots in urine, LCN2 has been proposed as an early marker of bladder, brain and endometrial cancer (Monier et al., 2000, 2002; Roy et al., 2008; Smith et al., 2008; Mannelqvist et al., 2012). Moreover, it was suggested to serve as a diagnostic marker for oesophageal and breast cancer progression or metastasis, gastric cancer and pancreatic tumors when measured as an individual biomarker or in a complex with other proteins (Kubben et al., 2007; Zhang et al., 2007; Moniaux et al., 2008; Yang et al., 2009; Du et al., 2011; Ören et al., 2016). In renal and thyroid solid tumors, LCN2 showed a positive correlation with malignant phenotypes (Iannetti et al., 2008; Barresi et al., 2010, 2012a,b). In ovarian solid tumors, LCN2 was associated with cancer differentiation, whereas LCN2 was also proved to be correlated with hepatocarcinoma and lung tumor progression (Friedl et al., 1999; Zhang et al., 2012). Even though LCN2 is suggested to have a role in the early stages of tumor development or as a prognostic marker, there is an urgent need for elucidating the role of LCN2 by studying its effects on metastasis behavior. Of course, the multitude of pathological events (cancer, inflammation, metastasis, organ failure) and affected organs (kidney, liver, heart, brain, pancreas, muscle) that give rise to alterations in LCN2 serum or urine levels may further require the definition of clear cut-off values that are indicative for a specific disease.

## Hepatic LCN2 expression

**Figure 3:**
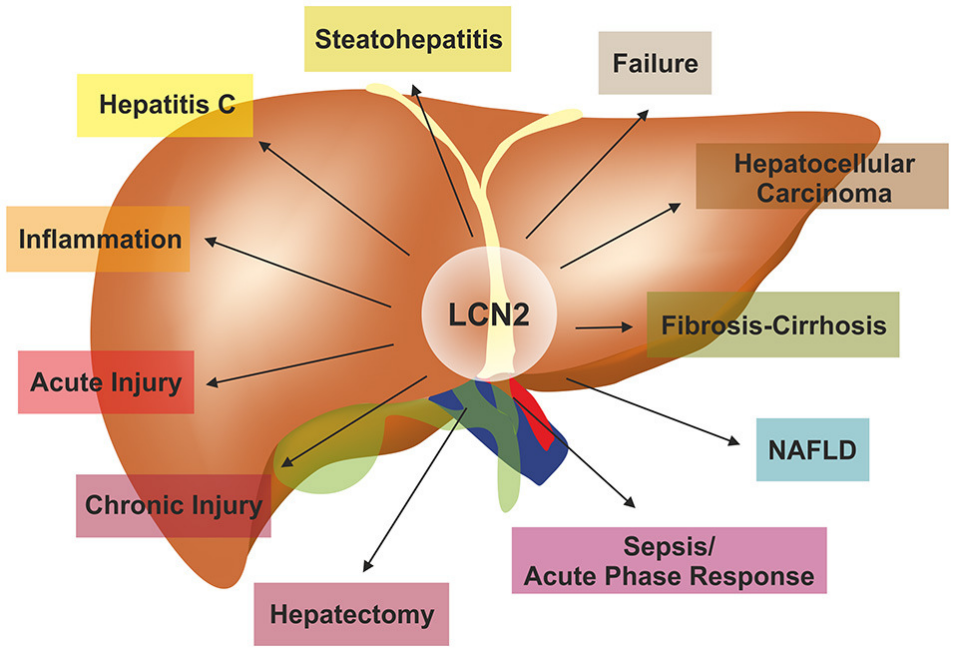
**LCN2 in the pathogenesis of liver diseases**. LCN2 is a versatile adiponectin that influences all kinds of liver diseases. For more details on experimental and clinical findings associated with altered LCN2 expression refer to Table 3.

During the last years, LCN2 has been studied widely as a possible non-invasive biomarker for several organ disorders, as outlined above. Hepatic expression of LCN2 in disease is the most intensively studied chapter with almost 1/4 of the published findings targeting the roles of LCN2 in liver homeostasis. The liver is the vital organ for fat and carbohydrate metabolism. It is the body's production facility for many metabolites, hormones, and proteins that also detoxifies substances and excretes metabolites. Thus, any functional impairment in liver affects undesirably the whole organism. Experimental and clinical findings indicating altered hepatic LCN2 expression in numerous disease conditions are given in Figure 3 whereas the most recent findings are gathered in Table 3.

**Table 3:** **Selected experimental and clinical findings associated with altered LCN2 expression in liver**.

**Species**	**Model/disease**	**LCN2 detection method**	**LCN2 expression**	**Finding**	**References**
Rat	Acute and chronic liver injury (CCl_4_, bile duct ligation)	IHC, WB, qRT-PCR	Elevated expression of LCN2 in hepatocytes after injury	LCN2 overexpression can indicate liver damage. Suggested as a biomarker of early hepatic injury	Borkham-Kamphorst et al., 2011
Mice (WT and *Lcn2*^−/−^)	Acute liver injury (LPS, ConA, bile duct ligation)	ELISA, WB, qRT-PCR	Liver injury induced LCN2 upregulated expression	LCN2 is a trustworthy biomarker of hepatic damage and serves possible protective role in liver	Borkham-Kamphorst et al., 2013
*Lcn2*-deficient mice	Hepatic inflammation (LPS)	NA	NA	LCN2 is an essential factor for regulation of inflammatory process manifestations during liver injury	Labbus et al., 2013
Human	Hepatic cirrhosis	ELISA	Increased expression in patients	Urine LCN2 is a prognosis marker of cirrhosis	Ariza et al., 2016
Rat	Chronic rejection	Mass spectrometry	LCN2 elevation in the chronic rejection group	LCN2 potential prognostic markers for predicting chronic rejection after liver transplantation	Wei et al., 2015
Human	Chronic HCV-induced fibrosis	ELISA, WB, Gelatin zymography	Urine LCN2 levels higher in patients with fibrosis/cirrhosis in the same line as MMP-9/MMP-2 ratio	Urinary LCN2 is a novel marker of hepatic fibrosis by reflecting urine MMP-9 activity in chronic hepatitis C	Kim et al., 2010
Human	Acute liver failure and chronic liver failure	ELISA	Baseline LCN-2 serum concentrations were significantly increased among acute liver failure patients as compared with chronic hepatic failure	LCN2 useful to discern acute from chronic hepatic failure and to monitor severity of the disease	Roth et al., 2013
Human	Chinese patients with NAFLD	ELISA	Circulating LCN2 elevated in patients compared healthy controls	Serum LCN2, correlates with inflammation and insulin resistance, and is correlated with disease progression	Ye et al., 2014
Human	Obese women with NAFLD	ELISA	Liver LCN2 protein and gene expression were higher in NAFLD than obese women without NAFLD	LCN2 related to NAFLD and liver damage	Auguet et al., 2013
Human	NAFLD patients	ELISA	Urinary LCN2 levels correlated with body mass index, glucose, and insulin levels in patients with steatosis; LCN2 levels correlated also with fibrosis stage and cirrhosis	LCN2 has a novel association between urinary levels and hepatocellular injury in these patients	Tekkesin et al., 2012
Mice (WT and *Lcn2*^−/−^)	High fat diet induced-NAFLD	WB	The molecular disruption of *Lcn2* in mice resulted in significantly potentiated diet-induced obesity, dyslipidemia, fatty liver disease, and insulin resistance	LCN2 contributes in regulation of lipid metabolism and insulin resistance	Guo et al., 2010
Mice (WT and *Lcn2*^−/−^)	High fat diet induced-obesity	NA	LCN2 supports function of PPAR-γ ligands	LCN2 regulates hepatic lipogenesis by affecting PPAR- γ expression	Jin et al., 2011
Rats	High fructose diet-induced NAFLD	WB, qRT-PCR, IF, ELISA	Overexpression of LCN2 in NAFLD mice	LCN2 to be correlated to inflammation, mitochondrial malfunction, oxidative stress and liver protective qualities	Alwahsh et al., 2014
Mice (WT and *Lcn2*^−/−^)	MCD diet-induced-NASH	WB, qRT-PCR, IF	Overexpression of LCN2 in NASH mice; LCN2 regulates PLIN5 expression in hepatocytes	LCN2 regulates liver lipid homeostasis by partially regulating PLIN5 expression	Asimakopoulou et al., 2014
Mice	Genetically induced NASH with fatty liver Shionogi mice	qRT-PCR, IHC	NASH mice presented higher LCN2 expression	LCN2 has a key role in NASH development	Semba et al., 2013
Human	Alcoholic fatty liver disease (AFLD) patients	ELISA	Increased hepatic LCN2 in AFLD patients compared to patients with alcoholic cirrhosis or simple steatosis	LCN2 drives ethanol-induced neutrophilic inflammation and participates in the development of AFLD; pharmacological neutralization of LCN2 is a potential treatment	Wieser et al., 2016
Mice (WT and *Lcn2*^−/−^)	MCD diet-induced-NASH	WB, qRT-PCR, IF	LCN2 elevated levels after MCD diet for 4 weeks induced intense leukocyte recruitment	LCN2 has an hepatoprotective role as it is required in neutrophil trafficking to liver	Asimakopoulou et al., 2016
Human	Patients who underwent curative resection of HCC	IHC	The expression levels of LCN2 and its receptor were both up-regulated in HCC tissues; high expression correlated with shorter overall survival	Expression of LCN2 and its receptor might serve in HCC prognosis and therapy	Zhang et al., 2012
Human	HCC patients	ELISA	Increased LCN2 expression in patients with HCV and HCC	LCN2 can be used as a future diagnostic marker with better sensitivity and specificity than MMP-9 for the progression of HCC	El Moety et al., 2013
Mice (WT and *Lcn2*^−/−^)	Bacterial infection or partial hepatectomy	ELISA	*Lcn2*-deficient mice demonstrated increased susceptibility to infection and reduced liver regeneration after hepatectomy	Hepatocytes are the major cell type responsible for LCN2 production after bacterial infection and hepatectomy; hepatocytic-derived LCN2 has important hepatoprotective roles in those conditions	Xu et al., 2015

*ConA, Concanavalin A; ELISA, Enzyme-linked immunosorbent assay; HCC, hepatocellular carcinoma; IF, immunofluoresence; IHC, immunohistochemistry; LPS, lipopolysaccharide; MCD, Methionine-choline-deficient diet; NASH, non-alcoholic steatohepatitis; PLIN5, Perilipin 5; PPAR- γ, Peroxisome proliferator-activated receptor-γ; qRT-PCR, quantitative real time polymerase chain reaction; WB, Western blot*.

To sum up, alterations in LCN2 expression and functions are reported in many hepatic conditions, including fatty liver, hepatic inflammation, hepatitis C, hepatic regeneration, acute and chronic liver injury, cirrhosis, and liver failure. In the following we will discuss some of the most striking observations that were placed in context with LCN2.

## Hepatic injury—inflammation

Based on experimental models of hepatic injury in which mice received injections of carbon tetrachloride (CCl_4_) or rats were subjected to bile duct ligation, a marked increase in LCN2 expression was found (Borkham-Kamphorst et al., 2011). The increase of LCN2 expression was correlated with liver damage and was inducible in cultured hepatocytes by diverse inflammatory stimuli, suggesting that LCN2 is as an early biomarker of liver inflammation (Borkham-Kamphorst et al., 2011; Labbus et al., 2013). In line, Ariza and colleagues found in a cohort of more than 700 patients with cirrhosis that urine LCN2 was markedly increased in the patients when compared to healthy control samples, establishing urine LCN2 as a prognostic marker of cirrhosis (Ariza et al., 2016). Most interestingly, in this study it was demonstrated that the measurement of urine LCN2 had a better predictive power than the measurement of serum LCN2 in discriminating acute-on-chronic liver failure (ACLF) patients from cirrhotic patients with acute decompensation. The authors suggested that this is due to the fact that the association between urine LCN2 and ACLF was not exclusively related to kidney function and that further urine but not serum LCN2 is independently associated with ACLF.

Similarly, the quantities of LCN2 in fresh spot urine samples were found to correlate with fibrosis score and reflected the increased activity of matrix metalloproteinase-9 (MMP-9) in urine in patients suffering from chronic hepatic C infection (Kim et al., 2010). Unfortunately, this study correlated LCN2 levels only to the grade of fibrosis as assessed according to the METAVIR fibrosis score in only 18 patients that had no or mild fibrosis and 24 patients with significant fibrosis, while the amount of inflammation (i.e., the activity) within the diseased liver was not considered. In a subsequent study, that included a total of *n* = 192 patients with chronic liver diseases of any etiology and healthy controls showed that LCN2 is a reliable indicator of liver damage that is positively correlated with inflammation, but is not correlated with the degree of liver fibrosis *per se* (Borkham-Kamphorst et al., 2013). In line with these findings, it was proposed that LCN2 serum levels may be diagnostically useful to discern acute from chronic hepatic failure (Roth et al., 2013). However, a recent report showed that LCN2 in plasma and urine are both suitable as independent predictive factors of acute-on-chronic liver failure that correlates with liver failure and systemic inflammation (Ariza et al., 2016).

Compared to normal controls, the livers of LCN2 deficient mice were more injured when exposed to short term application of CCl_4_, LPS and Concanavalin A, or subjected to bile duct ligation. This was reflected by elevated levels of aminotransferases and increased expression of inflammatory cytokines such as IL-1β, IL-6, and TNF-α which in turn resulted in prolonged NF-κB activation and sustained activation of STAT1, STAT3, and JNK pathways in hepatocytes. Based on these findings, it was supposed that as a counterpart to all these observations LCN2 has also hepatoprotective roles in phases of acute liver injury (Borkham-Kamphorst et al., 2013). In the same study, it was noted that hepatocytes of mice that lack LCN2 showed lipid droplet accumulation and increased apoptosis giving a slight hint of LCN2 as a modulating factor in lipid metabolism. In the same study it was demonstrated that although patients with chronic liver disease exhibited significantly higher concentrations compared to healthy volunteers, the levels of LCN2 were not suitable to discriminate between non-cirrhotic and cirrhotic liver-diseased patients, suggesting that LCN2 levels provide no correlation to the degree of liver fibrosis but provide a significant positive correlation to inflammation.

## Fatty liver disease and steatohepatitis

Fatty liver disease (FLD) is the abnormal hepatic fat accumulation (steatosis) that can also progress to fat accumulation combined with inflammation in the liver (steatohepatitis). In principle, there are three types of fatty liver. First, non-alcoholic fatty liver disease (NAFLD), a condition with prevalence up to 1–3 in western societies, where liver accumulates fat reaching more than 5% of the total liver weight because of factors independent of excessive alcohol intake (Baumeister et al., 2008; Bellentani et al., 2010; Fotbolcu and Zorlu, 2016). NAFLD is not just a liver disease as it is associated with cardiovascular disease, chronic kidney disease, type 2 diabetes, obesity, and dyslipidemia (Fotbolcu and Zorlu, 2016). NAFLD can progress to non-alcoholic steatohepatitis (NASH) where lipid accumulation is accompanied by inflammation (steatohepatitis) that cause irreversible hepatic damage (Neuman et al., 2016). Second, alcoholic fatty liver disease (AFLD), the second most common fatty liver condition, is as the name reveals the toxic accumulation of fat as a result of long-term excessive alcohol consumption. AFLD appears with smaller prevalence than NAFLD, but in patients both conditions lead to hepatic structural changes, obesity, metabolic disorders, and abnormal glucose tolerance (Reddy and Rao, 2006; Kotronen et al., 2010; Lívero and Acco, 2016). The third most widespread form of FLD is the acute fatty liver of pregnancy, a life threatening complication, for both mother and fetus, which develops in the last months of pregnancy (Zhang Y. P. et al., 2016).

Although there is a high prevalence of all kinds of FLDs in modern societies, no reliable non-invasive method for disease assessment has been identified yet. Therefore, the histological examination of liver tissue after liver biopsy remains still the gold standard for evaluating the degree of hepatic necro-inflammation and fibrosis (Fierbinteanu-Braticevici et al., 2010). During the last years, the increase of the enzyme alanine transaminase (ALT) was commonly measured clinically as a non-invasive biomarker for FLD assessment. However, the specificity of this marker for FLD is rather low, because it is also indicative of hepatocellular abnormalities without distinction between damage and steatosis (Musana et al., 2004; Myers, 2009; Barr et al., 2010). There are numerous studies available that correlated LCN2 with obesity, insulin resistance and diabetes (Yan et al., 2007; Law et al., 2010) rendering LCN2 as a possible biomarker for assessment of FLD.

A study that investigated the development of NAFLD in Chinese subjects revealed a close correlation of serum LCN2 with the progression of the disease and the occurrence of insulin resistance (Ye et al., 2014). Similarly, the circulating levels of LCN2 and hepatic expression of LCN2 were significantly higher in female NAFLD patients than in women with severe obesity with non-significant liver disease (Auguet et al., 2013), again suggesting LCN2 as a good biomarker for assessment of NAFLD. This study also showed that the administration of TNF-α, IL-6, resistin, and adiponectin stimulated expression in HepG2 cells, confirming previous reports demonstrating that the expression of LCN2 is moderated by pro-inflammatory triggers (Borkham-Kamphorst et al., 2011). Moreover, in another study analyzing adult patients with NAFLD, urinary LCN2 levels correlated well with the body mass index, insulin resistance, and lipid profiles (Tekkesin et al., 2012).

In experiments in *Lcn2*-deficient mice fed with a high fat diet to induce experimental steatosis, it was shown that the lack of LCN2 expression significantly potentiated diet-induced obesity, dyslipidemia, FLD, and insulin resistance (Guo et al., 2010). In a subsequent study, it was demonstrated that LCN2 is a critical modulator of PPAR-γ activation at the level of the recruitment of coactivators/corepressors thereby impacting adipogenesis and lipogenesis in adipose tissue and liver (Jin et al., 2011).

**Figure 4:**
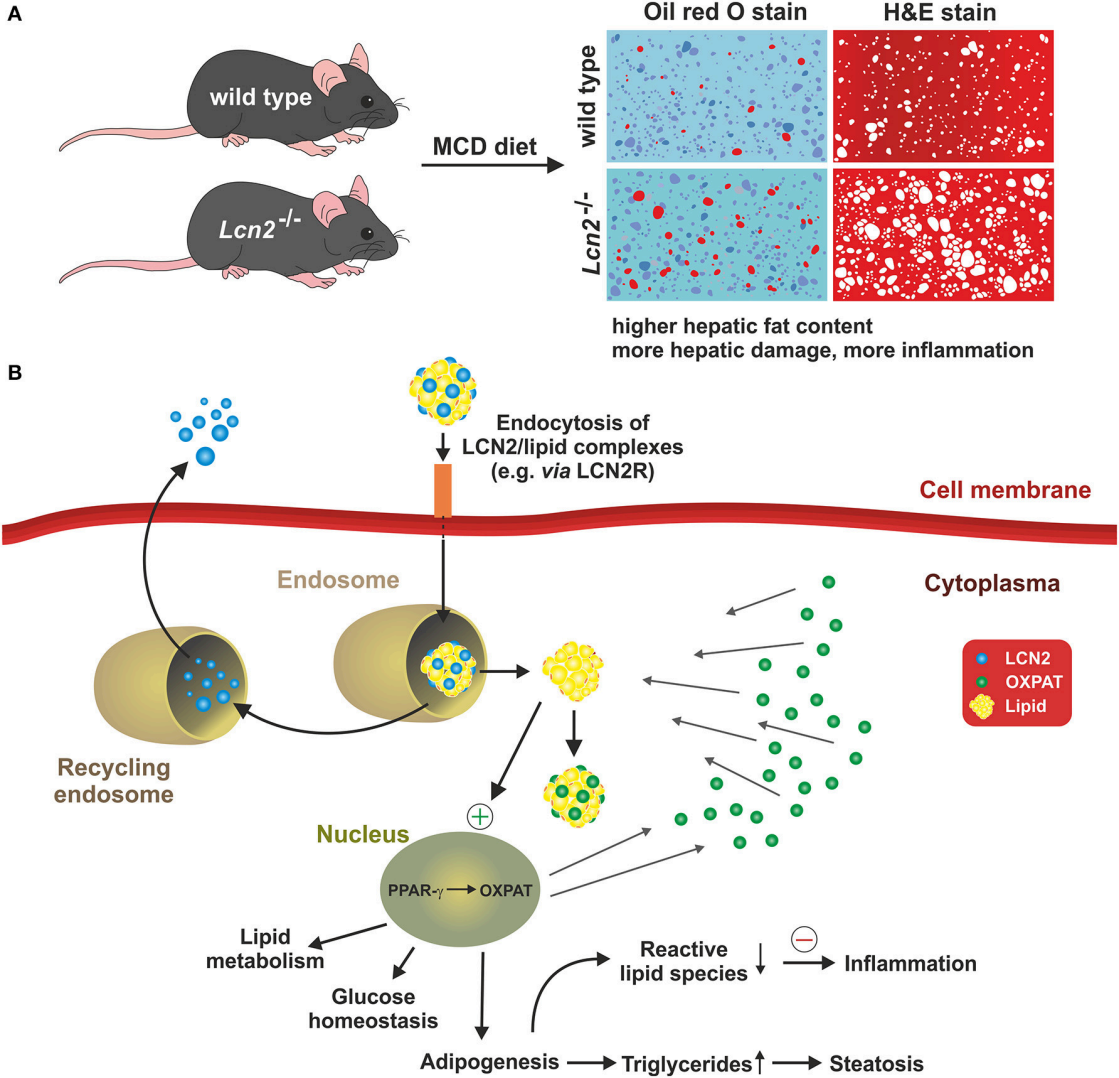
**LCN2 and fat metabolism**. A recent concept suggests that LCN2 is a key factor in controlling intracellular fat metabolisms in hepatocytes by regulating expression of the lipid droplet protein PLIN5/OXPAT. **(A)** The concept is majorly based on the finding that mice lacking LCN2 accumulate more lipids in the liver and show more hepatic damage and inflammation when fed a methionine-choline deficient diet (MCD) representing a nutritional model of NASH. **(B)** In the respective study, it was proposed that LCN2 imports lipids into hepatocytes either via specific receptors (e.g., LCN2R) or unknown endocytosis pathways. Within the cytoplasm, the LCN2/lipid complexes are first packed into endosomes whose slightly acidified microenvironment causes LCN2 to dissociate from the lipids. The lipids then move into the cytoplasm, where they are coated by PLIN5/OXPAT protecting them from intracellular degradation and oxidation. LCN2 may be recycled and secreted. PLIN5/OXPAT itself is up-regulated by PPAR-γ that is stimulated by the higher cytoplasmic fat content that is the consequence of facilitated lipid import by LCN2. Together, the suspected interaction of LCN2, PPAR-γ, and PLIN5/OXPAT presents a complex network that affects lipid metabolism, glucose homeostasis, and adipogenesis. In the presence of LCN2, intracellular concentrations of free reactive lipid species may be reduced and the overall inflammatory responses suppressed. Contrarily, in the absence of LCN2, the concentration of free fatty acids within the cytosol is increased predisposing for inflammation and steatosis.

The impact of LCN2 for the formation of obesity, inflammation, and obesity-associated metabolic dysfunction was also shown in rat models after feeding with a high fructose diet for 4–8 weeks. As in other models tested, the expression of LCN2 correlated with hepatic inflammation, mitochondrial malfunction, and oxidative stress (Alwahsh et al., 2014). We have demonstrated in a nutritional mouse model of NAFLD and in primary hepatocyte cell culture that LCN2 is directly linked to the intracellular formation and accumulation of hepatic lipid droplet accumulation partly via regulation of the lipid droplet protein Perilipin 5 (Asimakopoulou et al., 2014). Comparative analysis of wild type and *Lcn2* deficient mice revealed that the *Lcn2* lacking mice accumulated more lipids in their livers when fed a Methionine- and Choline-deficient diet (Asimakopoulou et al., 2014). Again, this finding indicates that LCN2 has an essential function in liver homeostasis and lipid metabolism (Figure 4).

**Figure 5:**
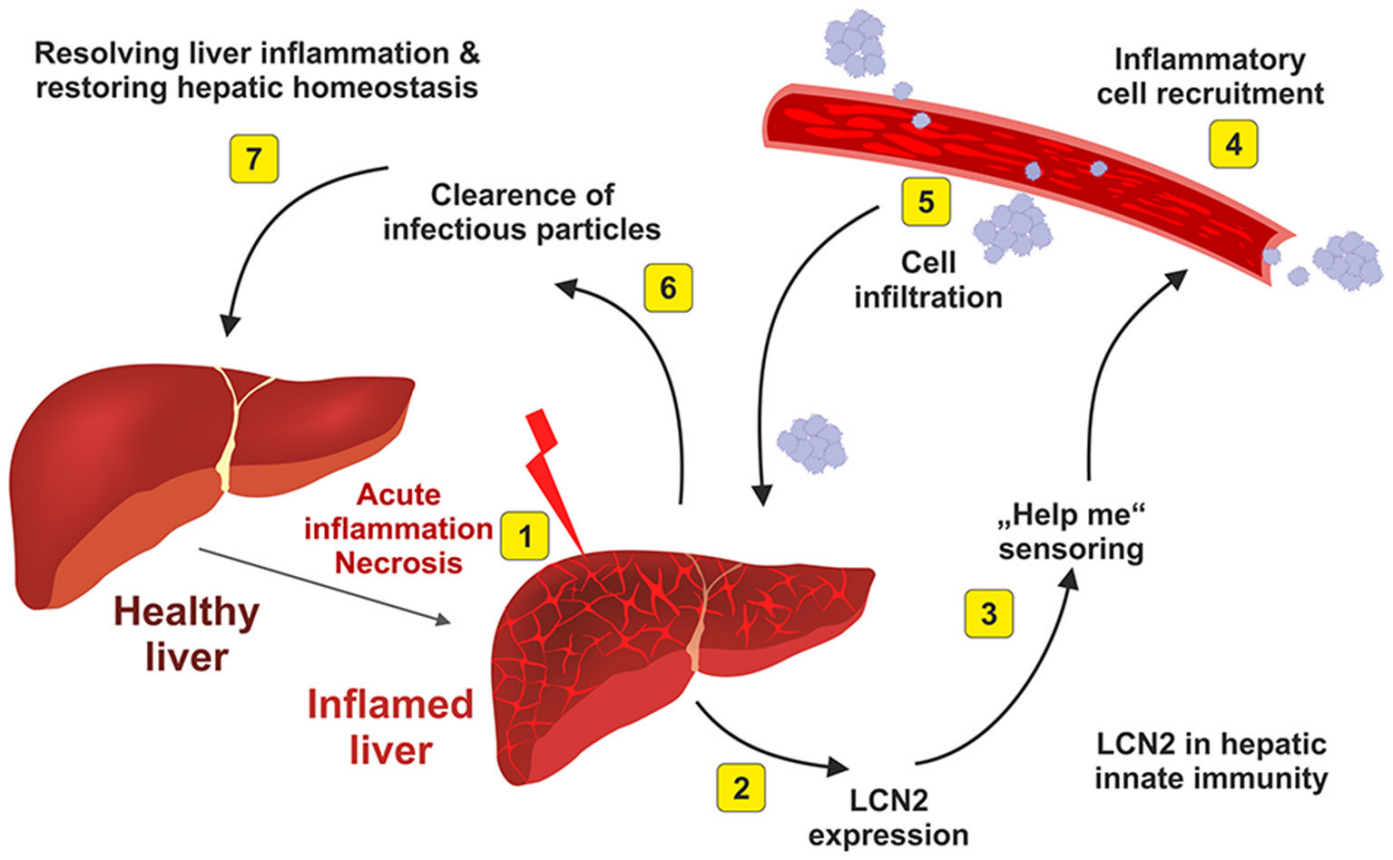
**Elevated hepatic LCN2 expression is a physiological indicator of hepatic inflammation**. After acute inflammatory stimulus (1), the liver synthesizes and secretes large quantities of LCN2 (2). This physiological response indicates that the liver is inflamed and needs help (3). As a consequence of the emitted “help me” signal, inflammatory blood cells that are necessary to begin the repair are recruited (4) and infiltrate the liver (5). The infiltrating cell populations help to destroy and clear infectious particles (6) and are helpful in restoring tissue homeostasis (7). For more details about this hypothesis are given elsewhere (Asimakopoulou et al., 2016).

Moreover, our study has shown that LCN2 is able to directly influence the expression of this lipid-droplet protein that is essential in maintaining the balance between lipogenesis and lipolysis and also of fundamental importance in fatty acid oxidation. Interestingly, Semba and colleagues found in the Fatty liver Shionogi (FLS) mice strain that are genetically programmed to develop NASH that LCN2 is directly linked to the pathogenesis of NASH. In addition, this study showed that LCN2 is primarily expressed by hepatocytes present in inflammatory cell clusters (Semba et al., 2013). Complementary data have suggested that the injury-induced upregulation of LCN2 is a kind of intrinsic “help me” signal (Figure 5) that might be relevant to recruit inflammatory cells into the injured tissue (Asimakopoulou et al., 2016).

After the finding that elevated LCN2 in circulation contributes to the progression of NAFLD (Ye et al., 2014), alcohol-related steatohepatitis was very recently shown also to closely relate to LCN2 levels (Wieser et al., 2016). In the respective study, the authors showed that *Lcn2*-deficient mice treated with ethanol presented less severe liver injury histologically and biochemically than the wild type mice. Reduced injury, according to ALT levels, was presented as well in wild type mice after LCN2 was neutralized before ethanol treatment with an LCN2 specific antibody. In addition, the control mice fed with ethanol showed increased *LCN2* expression that was mainly localized to leukocytes, especially neutrophils and to a lower content in monocytes and Kupffer cells (Wieser et al., 2016). Most interestingly, adoptive transfers revealed that neutrophil-derived LCN2 is critically involved in hepatic neutrophil immigration chronic alcoholic exposure, suggesting that the neutralization of LCN2 in alcoholic liver disease might be a promising therapeutic option to interfere with hepatic inflammation (Wieser et al., 2016). In parallel to this, we showed previously that during hepatic inflammation, the recruitment of leukocytes/neutrophils as indicated by CD45 mRNA content and Myeloperoxidase activity were significantly lower in *Lcn2* deficient mice compared to wild type mice (Asimakopoulou et al., 2016). In the same report we demonstrated that wild type mice after LPS treatment for 6 h recruited more Myeloperoxidase positive neutrophils when compared to LCN2 nulls, once again supporting the idea that LCN2 is critical for leukocyte and neutrophil recruitment (Asimakopoulou et al., 2016). The impact on migration and chemotaxis on polymorphonuclear neutrophils was previously demonstrated in mouse models in which the injection of LCN2 stimulated the mobilization of neutrophils (Schroll et al., 2012). All these data indicate that LCN2 acts as a paracrine chemoattractant that modulates neutrophil trafficking to the liver and other organs.

## Hepatocellular carcinoma and hepatitis C

**Figure 6:**
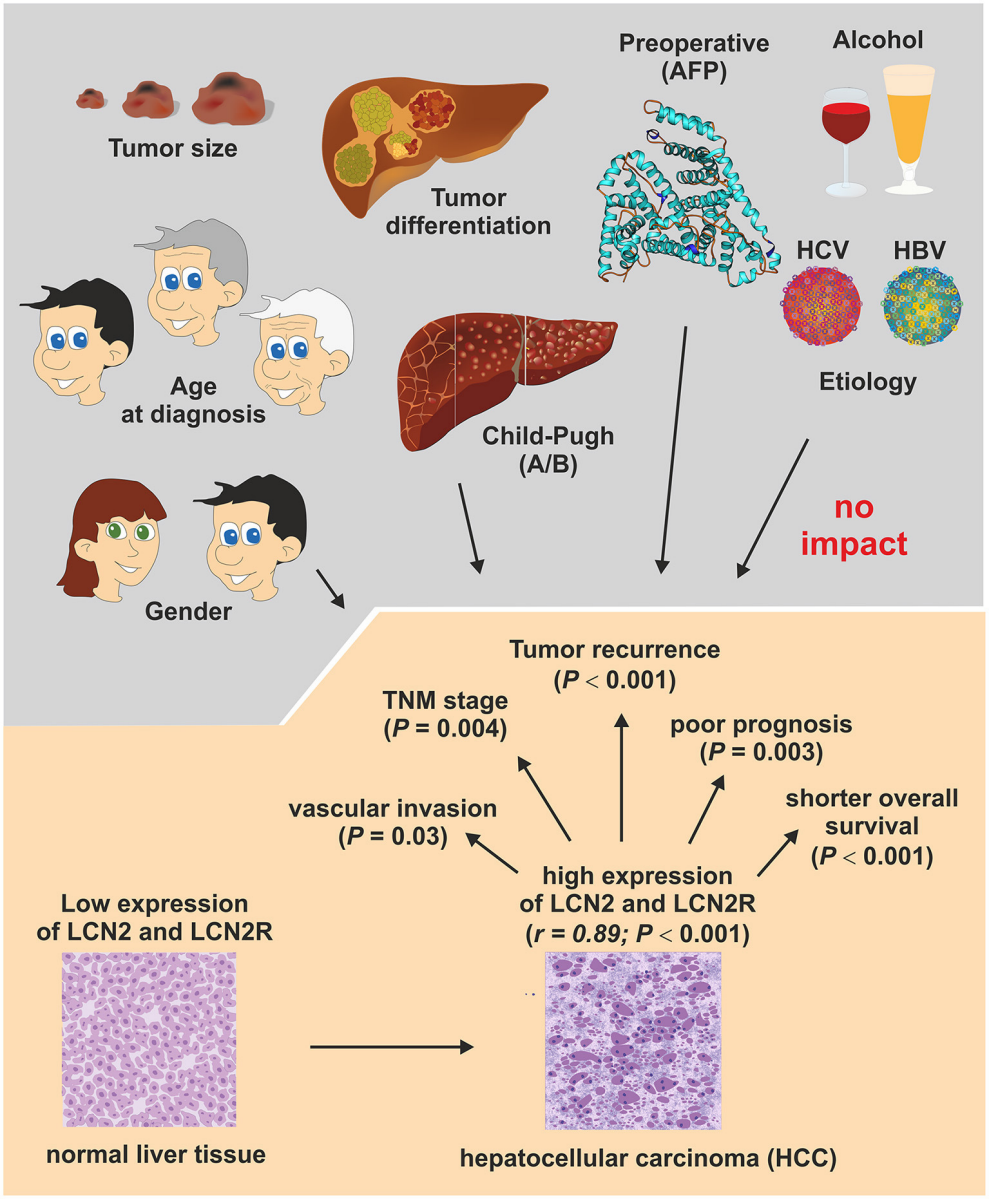
**Association of LCN2 and LCN2R expression with conventional clinicopathological HCC parameters**. An association study investigated the clinical significance of LCN2 and LCN2R in 138 patients who underwent curative resection of HCC. In conclusion, this study showed that the expression of both genes is correlated (*r* = 0.89; *P* < 0.001) and are up-regulated in HCC tissue and associated with vascular invasion status (*P* = 0.03) and TNM classification stage of malignant tumors (*P* = 0.004) that consider characteristics of the original primary tumor (T), the involved regional lymph nodes (N), and the occurrence of distant metastasis (M). In addition, the expression of both genes correlated well with tumor recurrence (*P* < 0.001), poor prognosis (*P* < 0.003), and overall survival rates (*P* < 0.001), suggesting that LCN2 and LCN2R expression are suitable prognostic factors and potential therapeutic targets in HCC. Details of this study are given elsewhere (Zhang et al., 2012).

Hepatitis C virus (HCV) infection is a major cause of chronic liver disease worldwide, leading to end-stage cirrhosis, and development of hepatocellular carcinoma (HCC). In 2010 urinary LCN2 levels were correlated to hepatic fibrosis and cirrhosis in HCV patients (Kim et al., 2010). Shortly after, an additional investigation demonstrated that LCN2 levels was elevated in HCC tissues and that both higher LCN2 and LCN2R expression correlated with shorter overall survival in HCC patients (Figure 6; Zhang et al., 2012).

Equally, LCN2 expression in patients with HCV and HCC is significantly increased (El Moety et al., 2013) proposing LCN2 as a reliable diagnostic marker for HCC progression. In this regard it should be mentioned that patients suffering from HCC are nowadays treated with hepatectomy, a surgical procedure of liver resection to remove hepatic tumors. This procedure is only possible because the liver is the only visceral organ that possesses a remarkable capacity to regenerate. In murine models of partial hepatectomy both Xu's and Kienzl-Wagner's teams demonstrated dramatic elevation of hepatic and serum LCN2 (Kienzl-Wagner et al., 2015; Xu et al., 2015). Xu et al. showed that the liver and specifically the hepatocytes are the main source of LCN2 responsible for the highly elevated expression of LCN2 after hepatectomy leading to a significant increase in circulating LCN2 levels for up to 24 h after hepatectomy (Xu et al., 2015). This finding indicates that LCN2 could serve as a biomarker for liver regeneration, however further experimentation is still necessary to elucidate the role of LCN2 in liver hepatectomy and regeneration.

## Diagnostic value of LCN2

In the reported findings described above, LCN2 is shown to be directly linked to several hepatic disorders in both humans and animals. Alterations in LCN2 expression are demonstrated in numerous models of hepatic pathogenesis conditions such as acute and chronic liver injury, inflammation, oxidative stress, mitochondrial dysfunction, bacterial infections, hepatitis C-induced fibrosis, liver failure, obesity, NAFLD, NASH, AFLD, HCC, and rejection after liver transplantarion (cf. Table 3).

Serum LCN2 could serve as a valuable biomarker of early stage hepatic damage (Borkham-Kamphorst et al., 2011, 2013). The measurement of LCN2 in urinary samples could be useful to predict the outcome of cirrhosis, liver transplantation rejection, and hepatic fibrosis (Kim et al., 2010; Wei et al., 2015; Ariza et al., 2016). Clinical data and *in vivo* models of FLD (NASH, NAFLD, AFLD) showed that elevated LCN2 levels are suitable to mirror hepatic lipid abnormalities, inflammatory response, mitochondrial malfunction, progress of FLD, and HCC (Guo et al., 2010; Zhang et al., 2012; El Moety et al., 2013; Semba et al., 2013; Asimakopoulou et al., 2014; Wieser et al., 2016). Since LCN2 is being proved to mediate hepatic function under multiple conditions, several studies during the last years have been focussed on the development of sensitive detection methods for LCN2. The mainly applied methods used for LCN2 detection include ELISA, Western blots and immunostainings. All these methods need specific LCN2 antibodies which have been available from a variety of pharmaceutical companies. We have previously listed the most common antibodies and systems used in research nowadays for the detection of LCN2 (Asimakopoulou and Weiskirchen, 2015). Most popular for quantification are diverse ELISA tests that have been proved to be the most sensitive and quick method to detect LCN2 elevation in early stages and conditions of experimental and clinical liver disease including FLD, NASH, and HCC.

However, there are actually many established biomarkers available for each of these diseases and it is too early to estimate the diagnostic and prognostic significance of LCN2 for daily clinical routine. It will be exciting to follow up if LCN2 will be added to the list of biomarkers suitable in HCC diagnosis and which superior insights in HCC development or progression will arise from LCN2 measurements that are not already disclosed by other serum markers such as α-fetoprotein (AFP), M39, M65, soluble CD163, or others that are already in use as tissue- or serum-associated HCC biomarkers (Waidmann et al., 2013; Morris et al., 2014; Chauhan and Lahiri, 2016).

## LCN2 in therapy

Many of the clinical conditions mentioned above that lead to hepatic malfunction or damage are associated with a significant upregulation of LCN2 expression in serum, urine, and/or liver tissue. Actually, there are many ongoing studies that analyse the precise molecular activities of LCN2 during disease progression. Recent studies performed in human kidney cells (i.e., HK-2) have shown that the application of recombinant LCN2 proteins induces excessive autophagy suggesting that LCN2 may have tremendous effects in AKI (Zhang W. et al., 2016). In contrast, protective effects of LCN2 were demonstrated in a mouse model of nephrotoxic serum nephritis (Eller et al., 2013). Likewise, a study that was conducted in myoblastic rat cardiomyocytes (i.e., H9c2) showed that the treatment with recombinant LCN2 inhibited autophagy (Chan et al., 2016). These contradictory findings indicate that LCN2 might have both protective as well as pathogenic activities. These double-edged biological properties may render therapeutic approaches complicated.

In regard to the liver, the chronic infusion of LCN2 exacerbated liver injury in two different models (i.e., MCD and high fat high cholesterol diet), while at the same time the observed hepatic injury in mice that were deficient for LCN2 was markedly reduced after feeding respective diets (Ye et al., 2016). Further analysis in this study showed that the inflammatory activities of LCN2 are mediated via induction of the chemokine receptor CXCR2 (formerly known as Interleukin 8 receptor, beta) that transduces its signals through a G-protein-activated second messenger system. Similarly, a pivotal and causal role of LCN2 was shown in cell models and animal models of AFLD (Cai et al., 2016). However, there are also other disease conditions in which this lipocalin protects the liver from acute liver injury (Borkham-Kamphorst et al., 2013), again demonstrating the versatile activities of LCN2.

Although it is now well-established that LCN2 has fundamental modulator activities during hepatic insult, there is still some work that remains to be done prior announcing LCN2 as a therapeutic target. Currently, initial studies that use anti-LCN2 antibodies (Tarín et al., 2016), siRNAs or shRNAs targeting LCN2 (Volpe et al., 2013; Miyamoto et al., 2016), specialized nanoparticle-based theranostic agents (Guo et al., 2016), or modulators that impacts LCN2 receptor expression (Cui et al., 2008) in different settings are initiated. In addition, the design of therapeutic small-molecule inhibitors for LCN2 was recently proposed (Suk, 2016). These molecules might be beneficial to target *de novo* LCN2 expression or its interaction with its cognitive receptors. Therapeutic strategies are nowadays tested in cell culture and preclinical models, but none of these has been tested in clinical studies so far.

## Concluding remarks

The lipocalin 2 protein was initially proposed to represent a secretory protein that is involved in the transportation of hydrophobic molecules, including steroids, retinoids, hormones, and lipids. Several years later, it has been promoted as a multifunctional siderophore that is involved in innate immunity by sequestering iron that in turn limits bacterial growth. A wide variety of studies have shown that the expression of this lipocalin is significantly altered in the pathogenesis of organ damage affecting kidney, heart, brain, pancreas, and lungs. In this review, we have summarized experimental and clinical findings linking LCN2 to liver disorders. It is obvious that LCN2 is significantly upregulated during phases of hepatic injury. In line with this assumption, numerous studies correlated LCN2 with general hepatic injury, inflammation, infection, cirrhosis, and fatty liver disorders. In all these pathological states LCN2 expression seems to be highly elevated, underpinning the notion that this lipocalin is a good biomarker candidate for a large variety of hepatic insults. Experimental findings have shown that the loss of LCN2 is correlated with a higher susceptibility to bacterial infections and more severe hepatic damage after challenge with appropriate hepatotoxins. Nowadays, several diagnostic test systems for detecting and quantification of LCN2 in urine, serum, plasma and tissue extracts are on the way to be incorporated into the routine diagnostics. Under some conditions, the measurement of urine LCN2 could more accurately reflect systemic inflammation or disease progression than serum LCN2. However, although the knowledge in LCN2 in liver biology has increased dramatically during the last years, there is still a mandatory need for studies that further characterized LCN2 and its pathways in the initiation, progression and potentially regression of hepatic lesions. These studies will potentially lead to novel therapeutic avenues or drugs that might be beneficial to interfere with progression or foster regression of inflammatory hepatic disease.

## Author contributions

RW and AA have written this review. SW prepared the figures. All authors agree to be accountable for the content of this work.

## Funding

RW is supported by grants from the German Research Foundation (SFB/TRR 57, P13/Q3) and a grant from the Interdisciplinary Centre for Clinical Research within the Faculty of Medicine at the RWTH Aachen University (IZKF Aachen, Project E7-6). None of the funding sources exerted influence on content or decision to submit the article for publication.

### Conflict of interest statement

The authors declare that the research was conducted in the absence of any commercial or financial relationships that could be construed as a potential conflict of interest.

## Abbreviations

AFLD: alcoholic fatty liver disease
AKI: acute kidney injury
ER: endoplasmic reticulum
FLD: fatty liver disease
HCC: hepatocellular carcinoma
HCV: Hepatitis C virus
LCN2: lipocalin 2
LPS: lipopolysaccharide(s)
MCI: mild cognitive impairment
MMP: matrix metalloproteinase
MS: multiple sclerosis
NAFLD: non-alcoholic fatty liver disease
NASH: non-alcoholic steatohepatitis
NF-κB: nuclear factor-κB
NGAL: neutrophil gelatinase-associated lipocalin.
